# Misrepresentation

**Published:** 1882-04

**Authors:** Ad. Lippe

**Affiliations:** Philadelphia


### MISREPRESENTATION.

AD. LIPPE, M. D., PHILADELPHIA.

The order of the day is misrepresentation. The eclectics of these latter days have no arguments to offer when they proclaim their liberty-professing excrescence of a revivified school of medicine, not only not in harmony with, but superior to, the healing art taught by Hahnemann; and this deplorable want of argument, with a deplorable want of facts on which to base any argument, has forcibly compelled them to resort to that detestable mode of defending a forlorn cause—misrepresentation.

It is not a pleasant duty to refer to this subject, but the times demand that perpetrators of this contemptible practice of misrepresentation should be exposed. In the March No. of this Journal we exposed the New York *Medical Times.* We have on several occasions alluded to the neglect of the American Institute of Homœopathy to contradict the misrepresentations to be found on page 801 of the second volume (Historical) of the Transactions of the "World's Homœopathic Convention," Philadelphia, 1876. The misrepresentation consisting in adding to the homœopathic colleges then existing, as enumerated by the President of said Convention in his address, another homœopathic college which was from its inception, until its final characteristic metamorphosis into an undertaker's establishment and a coffin factory, an out-and-out disreputable eclectic school, under the deceptive name of the Penn Medical University,

And now comes Dr. Richard Hughes, who presided over the "World's Homœopathic Convention," 1881; he there delivered an address full of misrepresentations, and now flushed by the apparent victory he gained in behalf of the eclectics over the true healers who honestly adhere to the teachings of the founder of a healing-art so ably misrepresented by Dr. Hughes, not knowing that another such apparent victory will prove itself to be his and the eclectics' Waterloo. In the January (1882) number of the British *Journal of* (what its editors, under a misrepresentation term) *Homœopathy*, and as part of the second Hahnemannian lecture, delivered at the London School of Homœopathy, by Dr. Hughes, on page 2, "The theory of the dynamization of medicines—*i. e.* the actual increase of power obtained by attenuation when accompanied by trituration and succession—is hardly propounded until the fifth edition (of the Organon). * * * On the other hand, there is the doctrine of a '*vital force*,' as the source of all the phenomena of life, as the sphere in which disease begins and medicines act * * * * But the earliest mention of this conception occurs in the fourth edition; and the full statement of it, with which we are familiar, in the fifth. Paragraphs 9—16 appear there for the first time."

In March, 1813, Hahnemann wrote the greatest of all his papers, "The Genius of the Homœopathic Healing Art," and republished it in 1833. In it we find the following passage: "The noxious influences which, as a general rule, create in us from without the various sicknesses, are generally so invisible and immaterial* that it is impossible for them to change or disturb the form and structure of the components of the body mechanically, nor can they bring into the circulation pernicious or acrid fluids whereby all our blood would be chemically changed or vitiated; an inadmissible, crude speculation of material brains which can in no way be proved."†

* Rare exceptions are some surgical conditions and complaints arising from indigestible foreign substances occasionally coming into the alimentary canal.

† Vide, The Organon, Vol. I, 248.

Hahnemann clearly defines in this sentence his definition of what he terms, in the sentence above, "a dynamical changed state of man"—a changed existence—called sickness. The influences causing disease are *immaterial*, that is his first proposition. Further on he says: "Therefore it becomes obvious that only the smallest doses become useful and necessary for a cure."* Hahnemann declares himself convinced both by his inductive method and by his experiments that the *smallest doses* only become useful and necessary, and he makes this declaration in 1813. Such *is* history; and the student who faithfully follows the great philosopher, to whom mankind forever will be indebted for his teachings, will be gratified to discover how this great man strove on, developing gradually the new healing art, never venturing to put on record his new progressive steps till he himself and the few of his followers who were led by him to make experiments, by the verification of his advancing discoveries had fully satisfied themselves of the correctness of his next forward step.

Why Dr. R. Hughes is so unwilling to follow the master, and why he continues to flood the journals and exhaust his eloquence by "*misrepresentations*" is a question open for debate; why he thereby leads others astray, and gives his endorsement to the growing tendency of our day to fill our journals, and even our Transactions with misrepresentations and falsifications of history we know not, but we do know that the motive must by necessity be a very bad one.

